# Purinergic receptors are a key bottleneck in tumor metabolic reprogramming: The prime suspect in cancer therapeutic resistance

**DOI:** 10.3389/fimmu.2022.947885

**Published:** 2022-08-22

**Authors:** Hamid Aria, Marzieh Rezaei, Shima Nazem, Abdolreza Daraei, Ghasem Nikfar, Behnam Mansoori, Maryam Bahmanyar, Alireza Tavassoli, Mohammad Kazem Vakil, Yaser Mansoori

**Affiliations:** ^1^ Noncommunicable Diseases Research Center, Fasa University of Medical Sciences, Fasa, Iran; ^2^ Department of Immunology, School of Medicine, Isfahan University of Medical Sciences, Isfahan, Iran; ^3^ Department of Laboratory Medicine, Faculty of Paramedical Sciences, Shahid Beheshti University of Medical Sciences, Tehran, Iran; ^4^ Department of Medical Genetics, School of Medicine, Babol University of Medical Sciences, Babol, Iran; ^5^ Department of Medical Genetics, Fasa University of Medical Sciences, Fasa, Iran

**Keywords:** therapy resistance, tumor microenvironment, metabolic reprogramming, purinergic receptor, cancer metabolism, immunometabolism

## Abstract

ATP and other nucleoside phosphates have specific receptors named purinergic receptors. Purinergic receptors and ectonucleotidases regulate various signaling pathways that play a role in physiological and pathological processes. Extracellular ATP in the tumor microenvironment (TME) has a higher level than in normal tissues and plays a role in cancer cell growth, survival, angiogenesis, metastasis, and drug resistance. In this review, we investigated the role of purinergic receptors in the development of resistance to therapy through changes in tumor cell metabolism. When a cell transforms to neoplasia, its metabolic processes change. The metabolic reprogramming modified metabolic feature of the TME, that can cause impeding immune surveillance and promote cancer growth. The purinergic receptors contribute to therapy resistance by modifying cancer cells’ glucose, lipid, and amino acid metabolism. Limiting the energy supply of cancer cells is one approach to overcoming resistance. Glycolysis inhibitors which reduce intracellular ATP levels may make cancer cells more susceptible to anti-cancer therapies. The loss of the P2X7R through glucose intolerance and decreased fatty acid metabolism reduces therapeutic resistance. Potential metabolic blockers that can be employed in combination with other therapies will aid in the discovery of new anti-cancer immunotherapy to overcome therapy resistance. Therefore, therapeutic interventions that are considered to inhibit cancer cell metabolism and purinergic receptors simultaneously can potentially reduce resistance to treatment.

## Introduction

Cancer is the second leading cause of mortality worldwide. In 2020, an estimated 19.3 million new cancer cases and 10.0 million cancer deaths are expected to be reported worldwide (1). Surgery, cytotoxic chemotherapy, radiation therapy, endocrine therapy, targeted therapy, and immunotherapy are the most common approaches in cancer treatments (2, 3). The immune system’s ability to recognize and, in some cases, successfully eliminate malignant cells has been demonstrated over the last century, leading to the development of various cancer immunotherapy strategies. One of these strategies is based on inhibiting immune checkpoints (ICPs). ICPs such as cytotoxic T-lymphocyte associated protein 4 (CTLA-4), programmed cell death 1 (PD-1), and programmed cell death ligand 1 (PD-L1) are immune system regulators that prevent the immune system from attacking cells. Some tumor cells can use these ICPs to escape immune response (4).

However, advancements in cancer treatment during the last decades had impressive effects; in the beginning, up to 90% of cancer-related deaths were caused by drug resistance and the subsequent inefficacy of treatment (5). Under the selective pressure of an immune response, cancer cells might develop distinct properties that enable them to escape detection by the immune system or even inhibit a functional immune response, resulting in tumor growth and relapse. So, resistance to conventional chemotherapeutic agents or innovative targeted medications remains a major issue in cancer treatment, accounting for most relapses and one of the leading causes of cancer death (6). A deeper knowledge of the mechanisms causing drug resistance is critically needed since it will contribute to establishing innovative therapeutic approaches that could improve clinical outcomes. On the other hand, metabolic reprogramming is required to accommodate the various demands of tumor cells during carcinogenesis, and recent studies have revealed its role in resistance to therapies. This review will describe how the specific receptors on the cell membrane, called purinergic receptors and its downstream signaling pathway, lead to therapy resistance by altering tumor cell metabolism. By reading this paper, readers will find out how purinergic receptors can provide the basis for treatment resistance with changes in tumor cell metabolism.

Each of the keywords purinergic receptors, therapeutic resistance, and cancer metabolism were searched once in pairs and once all in the Scopus, PubMed, and Google Scholar databases. The authors selected and studied the articles with titles that were exactly on these keywords. Any types of studies were included. The exclusion criteria were: articles published before 2010, unavailable articles, were not in English or were retracted. Duplicated articles are thrown out too. Then we summarize and integrate the findings from the papers into the full text as appropriate.

## Purinergic receptors

**Figure 1:**
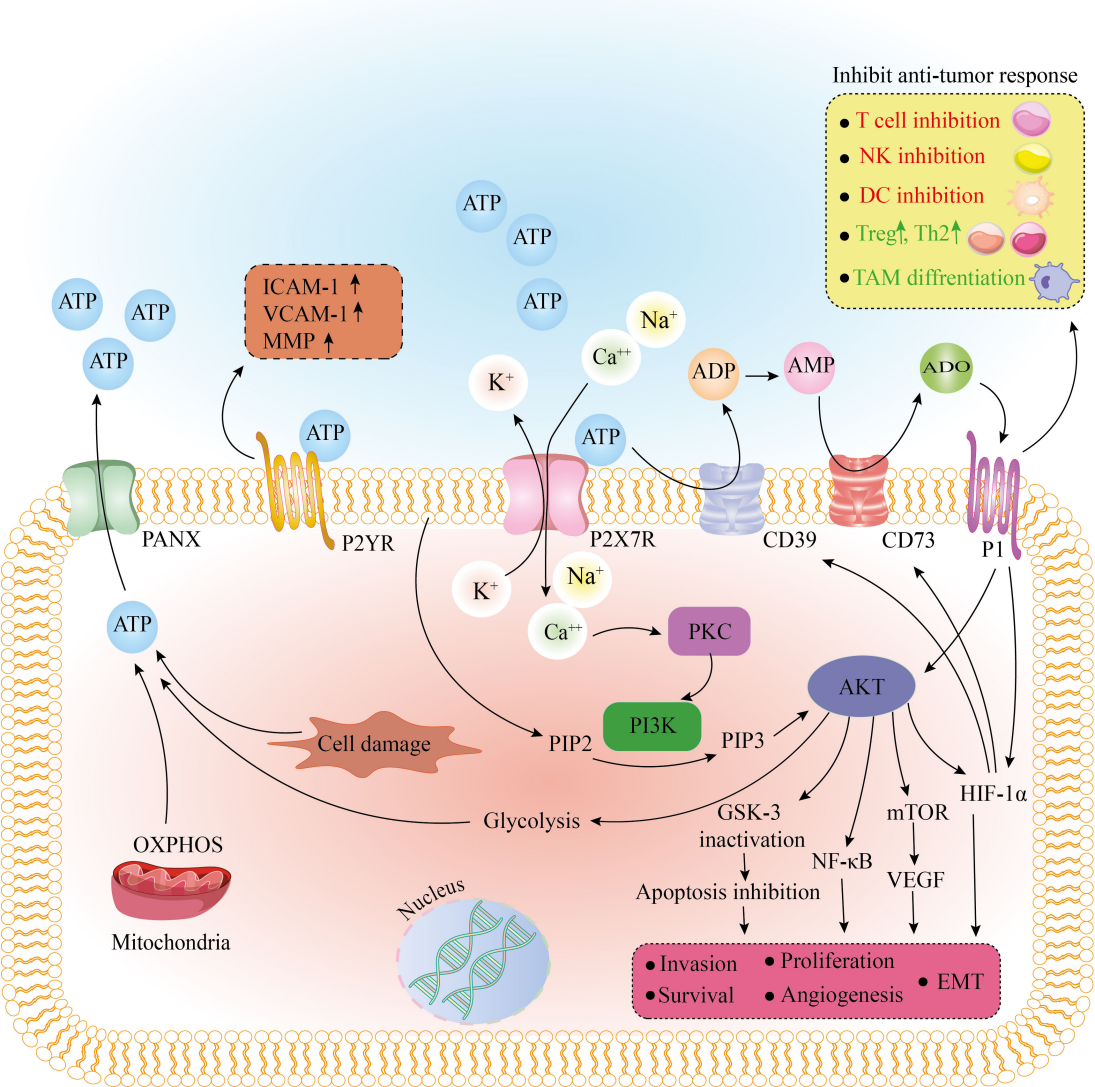
The effect of purinergic receptors on tumorigenesis and tumor cell metabolism. After binding to ATP, the purinergic receptor, P2X7R, removes K^+^ from the cell as Ca^2+^ and Na^+^ enter the cell. Also, extracellular ATP, due to the enzymatic function of CD39 and CD73, is converted to AMP and ADO. By binding to P1 receptors, ADO has tumor-promoting effects by inhibiting the immune response. Stimulation of the P1 receptor also causes activation of Akt and HIF-1a. An increase in intracellular Ca^2+^ activates PKC and PI3K, and PI3K by converting PIP2 to PIP3 activates Akt. As the main regulator, Akt activates HIF-1a, mTOR/VEGF, and NF-kb pathways and inactivates GSK-3, eventually leading to apoptosis inhibition, EMT, invasion, survival, and proliferation. Akt stimulates the glycolysis pathway. Glycolysis and OXPHOS or cell damage can produce ATP, leaving the cell through the PANX receptor. After binding to ATP, another purinergic receptor, P2YR, can increase ICAM-1, VCAM-1, and MMP, leading to adhesion and invasion of tumor cells. ADO, adenosine; AMP, adenosine monophosphate; ATP, adenosine triphosphate; CD39, cluster of differentiation 39; CD73, cluster of differentiation 73; DC, dendritic cell; EMT, epithelial-mesenchymal transition; GSK-3, glycogen synthase kinase-3; HIF-1a, hypoxia-inducible factor 1-alpha; ICAM-1, intercellular Adhesion Molecule 1; MMP, matrix metallopeptidase; mTOR, mammalian target of rapamycin; NF-kb, nuclear factor kappa B; NK cell, natural killer cell; OXPHOS, oxidative phosphorylation; PANX, pannexin; PI3K, phosphoinositide 3-kinases; PIP2, phosphatidylinositol-4, 5-bisphosphate; PIP3, phosphatidylinositol-3, 4, 5-triphosphate; PKC, protein kinase C; TAM, tumor-associated macrophage; Th2, T helper type 2; Treg, regulatory T cell; VCAM-1, vascular cell adhesion molecule 1; VEGF, vascular endothelial growth factor.

Cells respond to stress and damage (such as hypoxia) by releasing damage-associated molecular patterns (DAMPs), such as adenosine triphosphate (ATP). Extracellular ATP (eATP) can function as a “find me” signal for immune system cells and attract them to the location of the tissue damage (7). ATP can also be converted to adenosine (ADO) in two steps by CD39 and CD73 activity. ATP, uridine triphosphate (UTP), adenosine diphosphate (ADP), uridine diphosphate (UDP), and ADO are cellular mediators that have specific cell membrane ligands named purinergic receptors. Purinergic receptors and ectonucleotidases regulate various signaling pathways that play a role in physiological (transmitter and neurotransmitter) and pathological processes. Purinergic receptors are divided into three groups. Purinergic P0 receptors (P0Rs), purinergic P1 receptors (P1Rs), and purinergic P2 receptors (P2Rs): P0 responds to adenine, P1 (A1R, A2AR, A2BR, and A3R) responds to ADO, and P2 responds to ATP, ADP, UTP, and UDP. P2 is classified into two subfamilies: P2X, which are ion channels created by complexes of subunits (P2X1-P2X7) that stimulate fast depolarization coupled with Ca^2+^ and Na^+^ entry, as well as K^+^ export (**Figure 1**) (8), and P2Y, that have eight subtypes P2Y1, P2Y2, P2Y4, P2Y6, and P2Y11-14. ATP can activate P2Rs or be hydrolyzed by ectonucleotidases. Ectonucleoside triphosphate diphosphohydrolases (NTPDases, such as CD39), ectonucleoside pyrophosphatase/phosphodiesterase (ENPP), ecto-5’-nucleotidase (CD73), and alkaline phosphatases (AP) are the four groups of these enzymes (9). These enzymes create extra ligands for P2Y receptors in addition to restricting ATP signaling.

## Purinergic receptors and cancer development

Intracellular ATP is released *via* the pannexin-1 (PANX1) and P2X7 receptor (P2X7R) channels into the extracellular space (10). eATP levels are 103 to 104 times greater in different cancer types than in normal tissues (11, 12). eATP has an important role in cancer cell survival, growth, and resistance. It was demonstrated that eATP internalizes the cell and increases intracellular ATP, leading to cancer cell survival and drug resistance (13, 14).

On the other hand, as mentioned above, ATP as a DAMP can play a role in the activation and maturation of tumor-specific dendritic cells (DCs), in a process called immunogenic cell death (ICD), along with other factors (15). The ICD process leads to the induction of a tumor-specific and long-lasting acquired immune response (16). This issue can indicate the role of ATP as an anti-tumor and treatment resistance reducer. Whether ATP reduces or increases the resistance to treatment can depend on its concentration, tumor stage and the receptor binded to it.

The substantial amount of ATP and ADO in the TME activate purinergic receptors, including P2X7R (17, 18). When P2X7R is activated, the nucleotide-binding and oligomerization domain (NOD)-, leucine-rich repeats (LRR)- and pyrin domain-containing protein 3 (NLRP3) inflammasome is assembled, and pro-inflammatory cytokines including interleukin-1 (IL-1) and IL-18 are released (19). Moreover, the C-terminal domain of the P2X7R is involved in signal transduction and pore formation, while the N-terminal domain could influence immune cell sensitivity to external nicotinamide adenine dinucleotide (NAD^+^) and ATP (20, 21). When the P2X7R is overstimulated, it causes a large membrane pore (megapore) formation in cooperation with PANX1, resulting in tumor cell apoptosis, while paradoxically, it can enhance tumor growth and is associated with a high tumor grade, proliferation, survival, and chemo-resistance (22–24), which is discussed below.

These contrasting effects of the P2X7R depend on its level of activation and maybe the cell types used as a model study. Also, the human P2X7R gene is highly polymorphic, and various receptor splice variants have been reported. They play different roles and make diverse outcomes in cancer. P2X7B variant has the protumoral effect as well as P2X7A while lacking the pore-forming cytotoxic activity as well as nfP2X7 (25, 26). The P2X7B and nfP2X7 variants appear to be more expressed than P2X7A on tumor cells and promote cell survival (26–28). The P2X7J variant seems to be over-expressed in cervical cancer and acts as a negative regulator of P2X7A by decreasing its cytotoxic activity (29). Finally, the P2X7-V3 variant acts as long non-coding RNA (lncRNA) and increases the proliferation of tumor cells (30).

In the P2X7R^-/-^ mice, tumor growth was increased and infiltrated immune cells in TME had an immunosuppressive phenotype, with decreased helper and cytotoxic T cells and more suppressor or regulatory T cells (Treg) populations (31). Moreover, in P2X7R^-/-^ or nlrp3^-/-^ mice, anti-cancer therapy was unsuccessful; it was revealed that P2X7R was associated with an anti-cancer response and the NLRP3 inflammasome activation (32). But, P2X7R blocking (A740003 as an antagonist) promotes the infiltration of CD4^+^ T cells and decreases the expression of CD73 and CD39, reducing TME immunosuppression (31). It seems these controversial results depend on TME ATP levels. In P2X7R^-/-^ mice, the ATP level decreased mostly due to a deficiency in immune cell ATP release and an increase in ATP degradation caused by infiltrating Tregs. But in the P2X7R blocking condition, due to decreased ectonucleotidase expression and increased ATP release from cancer cells, ATP levels in the TME remain unchanged.

CD39 and CD73 are expressed in immune, tumor, and stromal cells, and they regulate ATP and ADO levels in the TME by their enzymatic properties on the cell membrane, converting eATP to ADO in two steps (33). ADO is one of the most critical chemicals identified as a tumor-promoting factor that inhibits the function of anti-tumor immune cells and increases Treg numbers, with an immune evasion effect (**Figure 1**) (34). By increasing vascular endothelial growth factor (VEGF) release and, as a result, more angiogenesis, ADO increases cancer cell survival and proliferation (35, 36). As the essential immunosuppressive receptors, ADO receptors (A2AR and A2BR) are found in various tumor tissues and act as a promoter of cell growth (37). As mentioned above, eATP acts as a “find me” signal; this signal is reversed in the TME, owing to the conversion of eATP into ADO by the CD39 and CD73 (7). As a result, purinergic signaling determines the tumor and immune cell interaction outcome. Increased CD73 and CD39 in immune cells lead to decreased eATP and increased ADO production, resulting in an immunosuppressive environment.

ATP, through purinergic receptors, causes cancer cells to migrate and play a role in the epithelial-mesenchymal transition (EMT) in several cancer types (38). Highly metastatic breast cancer cell lines have been shown to release more ATP into the extracellular medium and lead to P2Y2R activation. As a result, they have more potential to migrate and invade through the mitogen-activated protein kinase (MEK)/extracellular signal-regulated kinases (ERK1/2) or β-catenin pathway (39–41). P2X7R activation causes cell migration, up-regulation of EMT-related genes, and down-regulation of epithelial cadherins (E-cadherins) in prostate, breast, and osteosarcoma cell lines, all of which are mediated through phosphoinositide 3-kinases (PI3K)/Akt phosphorylation and ERK1/2 pathways (**Figure 1**) (42, 43). Transforming growth factor-β1 (TGF-β1), a well-known EMT inducer, also has been shown to cause lung cancer cells to produce ATP and then activate P2 receptors, which mediated actin remodeling and cell migration (44). In a metastatic breast cancer cell line incubated with endothelial cells, P2Y2R activation enhanced intracellular cell adhesion molecule-1 (ICAM-1) and vascular cell adhesion molecule-1 (VCAM-1) expression, resulting in increased adhesion between cancer cells and endothelial cells as well as cancer cell metastasis (45). P2Y2R expression in breast tumor tissue is higher at the tumor’s invasive edge, and its activation by ATP increases matrix metalloproteinase (MMP) production in prostate cancer cells (**Figure 1**) (46, 47). In a mouse model, P2X7R blockade significantly reduces neuroblastoma bone marrow metastasis (48). Incubation of gastric or breast cancer cells with ADO increases EMT gene expression, associated with A2AR/A2BR activation and the Akt-mammalian target of rapamycin (mTOR) or adenylyl cyclase (AC)/protein kinase A (PKA)/cyclic adenosine monophosphate (cAMP) pathway (**Figure 1**) (49). Furthermore, overexpression of CD73 has been demonstrated to stimulate tumor cell migration, invasion, and adhesion (50).

## Cancer cell metabolism, purinergic receptors, and therapy resistance

Conventionally tumors were treated with the highest tolerated drug dosage, but it has recently been shown that this therapeutic approach puts persistent pressure on tumors, causing the selection and inducing phenotypes of highly acquired drug-resistant cancer cells. Therapeutic resistance is conferred by anything that limits drug availability near its target or inhibits the cell’s capability to respond to it. Anti-cancer drug resistance is caused by various mechanisms, including genetic mutations, epigenetic alterations, increased repair of DNA damage, alteration of drug target, cancer heterogeneity, senescence escape, EMT, and drug efflux (51).

Metabolic reprogramming is one of the cancer signatures that can be seen as a cause of malignant transformation or a result of that. When a cell transforms to neoplasia and then cancer, its metabolic processes undergo a series of changes due to increased energy requirements with limited availability of nutrients or oxygen. A unique harsh environment is established during cancer development, including low pH, hypoxia, oxidative stress, and nutritional pressure (52). Cancer cell progression and growth require the activation of the cell cycle and metabolic pathways to produce nucleotides, fatty acids (FAs), amino acids, and cell membrane components (53). The modified metabolic environment of the TME can cause immune cells to undergo metabolic reprogramming, impeding immune surveillance and promoting cancer growth (54).

Despite promising advances in immunotherapy (such as immune checkpoint inhibitors, ICPI), the metabolically immunosuppressive TME is still a significant hurdle to tumor immunotherapy effectiveness (55). Tumor cells constantly adapt their nutrient uptake and metabolism to maintain proliferation, putting metabolic stress on infiltrating immune cells, and influencing antigen presentation, leading to immunosuppression and immune escape (56–59). Numerous researches have provided information on the involvement of mitochondria in treatment resistance. On the other hand, ICPI’s drug resistance is thought to be related to altered metabolic reprogramming of the immunosuppressive TME (58, 60).

Also, metabolic reprogramming is crucial for cancer metastasis and therapeutic resistance. Immunological cell infiltration and anti-tumor immune responses can be inhibited by immunosuppressive metabolites produced in the TME. As a result of the association between immune response and cancer metabolism, combination therapies that target ICPs and metabolic pathways could improve anti-cancer therapy effectiveness and overcome immunotherapy resistance. So, comprehensive knowledge of the metabolic variations between normal and cancer cells, as well as their effect on the anti-cancer response, will not only facilitate extending therapeutic alternatives but also combat therapy resistance. Effective therapies that target dysregulated metabolic checkpoints have the potential to remodel the immunological state of the TME, modulate the activation of T cells, improve the immunogenicity of cancer cells, and increase the efficacy of ICPI synergistically.

The purinergic receptors such as P2X7R are essential in metabolic disorders and cancer metabolic reprogramming (61). On the other hand, uncontrolled growth of cancer cells requires highly cellular energy which can be provided by purinergic receptors (62). Non-small cell lung cancer (NSCLC) cell line was found to have the ability to internalize highly concentrated eATP, which promoted intracellular energy supplement and enhanced growth, survival, and EMT (14). P2X1R and P2X7R inhibition reduce mitochondrial activity, calcium level, and cell proliferation in THP-1, Jurkat, HL-60, and U-937 cells. Furthermore, K^+^ is released through P2XR into the TME by necrotizing tumors, causing effector T cell suppression *via* autophagy and caloric limitation, which drive epigenetic and metabolic reprogramming (8, 63, 64).

Both cancer and immune cells have similar metabolic characteristics, relying on glycolysis to achieve the energy needed for fast proliferation. So, the TME has an exceptionally high demand for nutrition (glucose, amino acid, FA), and competition between immune and cancer cells can decrease the anti-cancer response (65). The researchers found that intracellular ATP levels were important in multiple drug resistance (MDR); compared to their parental cells, chemo-resistant cancer cell lines had higher intracellular ATP levels. They also demonstrated that delivering liposome-encapsulated ATP to drug-sensitive cells artificially leads to drug resistance while using a glycolysis inhibitor to deplete intracellular ATP sensitized resistant cancer cells (66).

ATP-binding-cassette (ABC) transporters are transmembrane proteins that modulate the biodistribution of endogenous and exogenous products by translocating diverse substrates from intracellular to extracellular media. These transporters are involved in steroid production, immune responses, and reproductive barrier functions (67). ABC transporter subunits include P-gp (P-glycoprotein, or ABCB1), MRP1 (multidrug resistance protein 1, or ABCC1), and BCRP (breast cancer resistance protein, or ABCG2). They can efflux many drugs from the cell, reducing their interaction with intracellular targets and therapeutic efficacy. Overexpression of ABC transporters is one of the key chemo-resistance mechanisms in various malignancies (23, 68). Internalized ATP molecules by micropinocytosis improve ABC transporter expression and then efflux tyrosine kinase inhibitors (TKIs) and chemotherapy drugs, leading to less drug accumulation and enhanced cell survival. Reduced intracellular drug concentrations and increased ATP levels enhance ATP binding and decrease TKI binding on receptor tyrosine kinases (RTKs), resulting in enhanced RTK-dependent signaling and drug resistance. They postulated that ATP serves as an energy molecule that promotes drug efflux as well as a signal-transduction molecule that activates cell survival signaling pathways (13).

eATP may contribute to cancer treatment resistance through purinergic receptor signaling (69). By P2Y-mediated overexpression of MRP2 and drug pumping, researchers revealed that ATP increased chemo-resistance in colorectal cancer cells (70). Non-chemo-resistant patients had higher P2X7R levels and lower A2A levels in CD8^+^ T cells than chemo-resistant patients (71). Another study found that when ATP stimulated the P2X7R, it had an anti-apoptotic effect in melanoma cells treated with methoxyestradiol (72). eATP increases tumor cell survival and drug resistance by increased glucose transporter 1 (GLUT1) expression through the P2X7R-PI3K-Akt and hypoxia-inducible factor 1α (HIF-1α) (17).

Defects in the autophagy in tumor cells or purinergic receptors in immune cells result in a poor response to the drug (16, 73). Immune cells, dying or stressed cells of TME, can release eATP, potentially resulting in an ATP-rich, tumor-friendly environment (74). eATP plays a role in TME immunomodulation and may impact therapeutic outcomes. NSCLC cell line, through high ATP internalization, promotes intracellular energy supplement and enhances therapy resistance (14). Signaling mediated by ADO derived from eATP would result in the formation of an immunosuppressive environment and therapy resistance (34).

But in a contradictory way, a recent study found that ATP-decorated and doxorubicin-loaded silica had a considerable anti-tumor effect against doxorubicin-resistant murine lymphoma. The nanocomposite increased apoptosis by activating the P2X7R (75). The noteworthy point in this context is that long-term activation of P2X7R by high levels of eATP can result in the formation of a macropore, which causes cell death by the plasma membrane depolarization. Compared to levels found in normal tissues, the TME has higher eATP (76), but these concentrations are probably not obtained due to ectonucleotidases. It seems there will be a concentration window where eATP concentrations are low enough to avoid significant drug resistance but high enough to promote anti-cancer immune responses (51). Hence P2X7R can either promote cell survival or cause cell death depending on its activation state.

### Glucose metabolism

**Figure 2:**
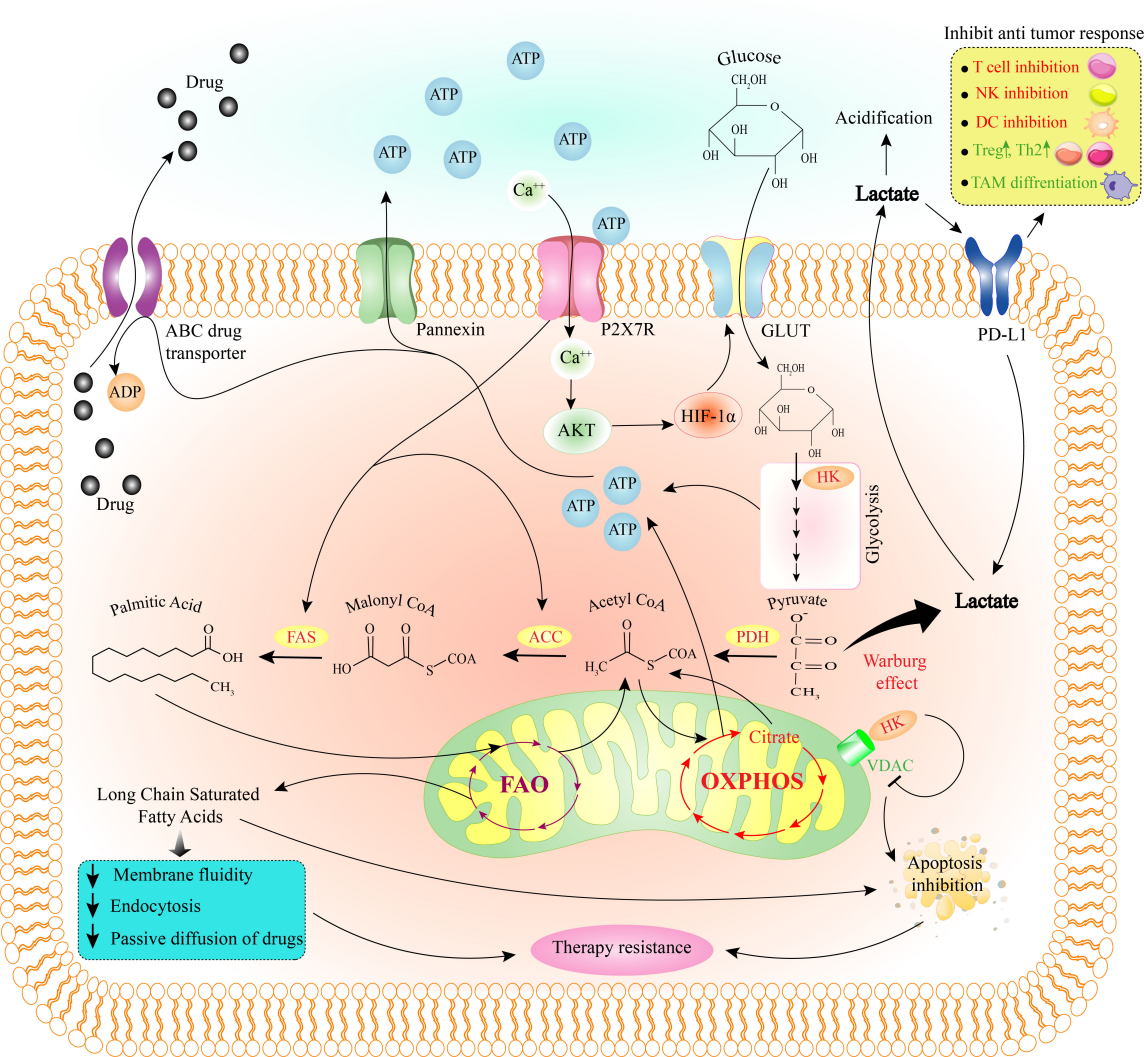
How purinergic receptor activity induces therapy resistance through metabolic reprogramming in the tumor cell. Due to the activity of the P2X7R and the increase of intracellular Ca2+, Akt and then HIF-1a is activated. HIF-1a stimulates GLUT to allow glucose to enter the cell and trigger the glycolysis pathway. HK, one of the enzymes in the glycolysis pathway, can inhibit tumor cell apoptosis by binding to VDAC on mitochondria and inhibiting it. Pyruvate from the glycolysis pathway is mainly converted to lactate through the Warburg effect. After leaving the cell, lactate can inhibit the anti-tumor immune response by acting on PD-L1. On the other hand, some pyruvate with the effect of PDH, ACC, and FAS eventually convert to fatty acids (palmitic acid). P2X7R activity also stimulates FAS and ACC. Then the fatty acid produced in the cytoplasm enters the mitochondria and the FAO pathway and produces long-chain saturated fatty acids. By reducing membrane fluidity, endocytosis, passive diffusion of drugs, and apoptosis, long-chain saturated fatty acids cause tumor cell therapy resistance. Also, ATP produced through glycolysis and OXPHOS help the drug export *via* ABC transporter. ABC, ATP-binding cassette; ACC, acetyl-CoA carboxylase; ATP, adenosine triphosphate; DC, dendritic cell; FAO, fatty acid oxidation; FAS, fatty acid synthase; GLUT, glucose transporter; HIF-1a, hypoxia-inducible factor 1-alpha; HK, hexokinase; NK cell, natural killer cell; OXPHOS, oxidative phosphorylation; PDH, pyruvate dehydrogenase; PD-L1, programmed death-ligand 1; TAM, tumor-associated macrophage; Th2, T helper type 2; Treg, regulatory T cell; VDAC, voltage-dependent anion channel.

In normal conditions, pyruvate enters the tricarboxylic acid cycle (TCA) and undergoes oxidative phosphorylation (OXPHOS) when the oxygen level is sufficient. In the lack of oxygen, glycolysis converts pyruvate to lactate (77). First, Warburg was shown that independent of the access to oxygen or the efficiency of mitochondrial OXPHOS; cancerous cells prefer to receive ATP from glycolysis, which is referred to as the “Warburg effect” or “aerobic glycolysis” (**Figure 2**) (78). Besides substantial energy, glycolysis provides pivotal intermediates such as nucleotides, amino acids, and lipids required for cancer cell biosynthesis (79).

Glycolytic-promoting factors including c-Myc, HIF-1, GLUT1, hexokinase (HK), pyruvate kinase (PK), phosphofructokinase (PFK), phosphoglycerate kinase 1 (PGK1), lactate dehydrogenase (LDH), and pyruvate dehydrogenase kinase isozyme 1 (PDK-1) that activated in tumor cells are described as tumor markers (80, 81). The higher glycolytic flux increases glucose absorption, glycogen synthesis, and lactate production. Lactic acid accumulation in the TME affects immune cell functions, impairs cell metabolism, proliferation, and activation, and decreases mobility and cytotoxicity (82–84). Furthermore, high lactate levels enhance Treg survival as well as a tolerogenic phenotype in DCs and macrophages, resulting in a more suppressor phenotype in TME while tumor cells are not targeted (**Figure 2**) (85, 86).

The pentose phosphate pathway (PPP) is another metabolic flux stimulated in malignant cells. The PPP increases the formation of 5-carbon sugars by degrading glucose. 5-carbon sugars are needed for nucleic acid synthesis, as well as the production of the oxidoreductases cofactor nicotinamide adenine dinucleotide phosphate (NADPH). NADPH is required for lipogenesis and keeping the antioxidant glutathione in reduced form (GSH) (87). Since cancer cells prefer aerobic glycolysis, which produces a lot of lactate and H^+^, the acidic TME facilitates rapid carbon incorporation into nucleotides, lipids, and amino acids and finally promotes cell proliferation (88, 89). As a result, blocking glycolysis as novel anti-tumor therapy can significantly decrease tumor cell proliferation and even play a role in tumor cell death (90).

In the TME, P2X7R stimulation by eATP has induced GLUT1 expression and enhanced aerobic glycolysis and OXPHOS through the PI3K-Akt pathway and HIF-1 signaling, which finally leads to increased ATP production (17, 91). Also, the intracellular HIF-1 was activated by stimulating the A2BR and Warburg effect (**Figure 1**) (92). Due to increased glucose transport and aerobic glycolysis in tumor cells, they have a higher intracellular ATP level than normal cells in the same tissue (14, 93). The P2X7R activation has downstream consequences similar to the Warburg effect, e.g., overexpression of PDK-1 (94). Amoroso et al. found in P2X7R-transfected neuroblastoma and HEK293 cell line an enhanced lactate production linked with cell proliferation, a characteristic of the Warburg effect (95).

In leukemia cells, increased ATP release was associated with increased mitochondrial quantity and activity, resulting in cancer cell proliferation (96). P2X7R can stimulate mitochondria by elevating the resting mitochondrial potential (ΔΨm) and mitochondrial calcium level, supporting the Warburg effect and proliferation (97). Furthermore, P2X7R enhanced O2 consumption and NADPH oxidase 2 and decreased respiratory rate, while its overstimulation leads to a decline in ΔΨm, as well as mitochondrial fragmentation and cell death (91, 95, 98, 99). P2X7R also reduced pyruvate dehydrogenase (PDH) activity, enhanced ERK phosphorylation, the PI3K/Akt/glycogen synthase kinase 3 (GSK3)/β-catenin, and mTOR/HIF1/VEGF signaling pathway (**Figure 1**) and increased the glycogen storage due to a decrease in GSK3 (100, 101). These metabolic changes are made to prevent aerobic responses (94, 95).

Cancer stem cells (CSCs) are the subpopulation of cancer cells with the ability to self-renewal and differentiation into any cell type. They have a role in recurrence, metastasis, heterogeneity, and drug resistance. With mitochondrial energy metabolism, CSCs absorb low-level FAs to produce ATP, NADPH, TCA intermediates, and nucleotide bases, which drive cancer cell survival and proliferation (102). The activation of the Akt pathway in CSCs causes the production of glycolysis enzymes such as PDK-1 and HK-1 (103). CSCs had lower glucose consumption, reactive oxygen species (ROS), intracellular ATP levels, and a preference for OXPHOS for energy supply (104). Many studies have shown that the P2X7R promotes CSC maintenance and asymmetric cell division (23, 105).

Because cancer cells have a high rate of glucose uptake and glycolysis, the competition for glucose suppresses calcium signaling, mTOR activity, glycolysis, and interferon (IFN)-production in T cells, leading to T cell exhaustion, reducing anti-cancer immunity, leading to immune evasion (106). An immunomodulatory receptor, PD-L1 overexpression suppresses the cytotoxic function of T-cells and enhances tumor cell resistance to lysis. According to Chang et al., increased PD-L1 on cancer cells increases mTOR function and glycolysis (**Figure 2**), leading to cancer-driven glucose limitation, changes CD8^+^ T-cell metabolism, and reduces T cell’s ability to produce IFN-γ (107). Antibodies that inhibit PD-1/PD-L1 or CTLA-4 impair tumor cell glycolysis and increase TME glucose levels and T cells glycolysis (106). By restricting the Ca2+-nuclear factor of activated T cells 1 (NFAT1) signaling pathway in CD4^+^ T cells, glucose deprivation additionally reduces anti-cancer activities of intratumoral T helper 1 (Th1) CD4+ T cells (108). A chemo-resistant stem-like side population in malignancies has increased glycolytic activity. This modulation is dependent on Akt pathway stimulation because of increased intracellular ATP concentration suppressing AMP-activated protein kinase (AMPK) (103). Overall, the findings suggest that tumor-derived glycolysis metabolites like lactate suppresses immune cell activity and leads to immunological evasion or therapy resistance (60, 109, 110). One of the mechanisms by which lactate reduces the effectiveness of immunotherapy is to increase the expression of PD-L1 (**Figure 2**) (111). Therefore, targeting LDH, which converts pyruvate to lactate, is one of the potential targets for overcoming treatment resistance (112).

P2Y1 receptors on vascular endothelial cells are activated by eATP and ADP, which transactivates VEGFR2 to induce angiogenesis (113, 114). In a recent study, Palinski et al. showed that extracellular vesicles from sarcoma patients enhance neo-angiogenesis *via* a P2X4R-dependent pathway. They also found that intracellular Ca^2+^ mobilization, mitochondrial activation, increased eATP, and lysosomal P2X4R trafficking to the cell membrane, all of which are essential for cell migration and vessel formation (115). In another study, Lapel et al. showed that glycolysis and OXPHOS are required for vasa vasorum endothelial cell (VVEC) angiogenic responses. The increased glycolytic activity was accompanied by increased PFKB3, HK, and GLUT 1 in VVEC after P2R agonists and ATP treatment. P2R agonists reduced PDH phosphorylation while increasing the expression of succinate dehydrogenase (SDH), cytochrome oxidase IV (COX IV), and F1F0-ATP synthase subunit. Furthermore, P2R stimulation caused an increase in mitochondrial Ca^2+^, showing that mitochondria are involved in VVEC angiogenic activation (116).

### Lipid metabolism

FAs uptake by tumor cells is very high, and their metabolism is crucial to the synthesis and maintenance of the cellular membrane, cell division, energy supply, and signaling pathway intermediates (117). In other words, tumor cell survival and metastasis are dependent on FAs absorption, consumption, and catabolism *via* the FA oxidation (FAO) pathway (118). Lipid accumulation leads to cancer cell survival and contributes to chemotherapy resistance, which is also significantly linked to CSCs in the tumor cell population (119). Citrate comes from glycolysis or glutaminolysis, exits mitochondria, and enters the cytoplasm, where ATP-citrate lyase converts it to the lipogenic substrate acetyl-CoA. Acetyl-CoA serves as a source of FAs, converted into phospholipids and triacylglycerols (120). FA synthase (FAS) is a central regulator of lipid metabolism that creates palmitate from the condensation of acetyl-CoA and malonyl-CoA (**Figure 2**). Overexpression of FAS has been associated with cancer growth, poor prognosis, and invasion in various cancers (121, 122), so it could be a potential candidate for anti-cancer drugs (123). One of the major components of membrane lipids is cholesterol, which plays a role in T-cell receptor (TCR) clustering and immunological synapses development (124). Because of an increase in the cell membrane cholesterol content of CD8^+^ T cells, suppressing cholesterol esterification resulted in potentiated effector activity and improved proliferation of CD8^+^ but not CD4^+^ T cells (125).

A group of studies links P2X7R activity to increased FA catabolic pathway and tumor cell plasticity (126). Furthermore, the loss of the P2X7R favored FAO and decreased ΔΨm, FA metabolism enzymes like acetyl-CoA carboxylase (ACC) and FAS. Also, loss of the P2X7R leads to higher serum cholesterol, triglyceride, lipid accumulation, adipocyte hyperplasia, obesity, and insulin resistance, all of which reduce proliferation, survival, and metastasis of tumor cells (127–129).

Lipid raft enrichment has been seen in prostate and breast cancer cell lines (130). The P2X7R isoforms cannot open the pore due to cholesterol in cell membranes (131). So, cholesterol-rich lipid raft regions are favored sites for P2X7R to preserve its ion channel activities just while avoiding apoptosis. In this way, lowering cholesterol or destroying lipid rafts causes cancer cell death (132).

On the other hand, high FAs can block the killing activity of T cells against tumor cells, allowing the tumor to escape the immune system, and lowering the efficacy of anti-cancer therapies that require a competent immune system (133). Lipid accumulation in tumor cells was associated with CSCs function and therapeutic resistance (119). FAS overexpression has been related to chemotherapy resistance in various tumors (121). Reducing low-density lipoprotein uptake decreases pancreatic adenocarcinoma’s oncogenic characteristics and makes cancer cells more susceptible to treatment (134). As a result, manipulating the lipid metabolism of tumor cells to increase immune function could be novel immunotherapy in the future.

### Amino acid metabolism

Critical amino acids such as glutamine are necessary for growth and proliferation because they provide both carbon and nitrogen for the biosynthesis of nucleotides (135). Enhanced glutamine metabolism is involved in different metabolic pathways like the Warburg effect in cancer cells. Glutaminase converts glutamine to glutamate, and glutamate is converted to α-ketoglutarate in the mitochondria by the glutamate dehydrogenase enzyme. α-ketoglutarate is a TCA cycle intermediate that serves as a substrate for the synthesis of NADH and oxaloacetate (136).

Because tumor cells require glutamine metabolism for survival, glutamine levels are associated with tumor sensitivity to anti-cancer treatments (137). Tumor cells consume glutamine and make it out of the T cell’s reach, preventing the T cells from proliferation and anti-tumor activity (138). According to a recent study, glutamine metabolism in tumor cells reduces the effectiveness of anti-tumor immune responses (139). Myeloid−derived suppressor cells (MDSCs) are immunosuppressive cells that are activated and increased in response to several growth factors and cytokines released by cancer cells (140). MDSC apoptosis is induced by glutaminase inhibitors, making ICPI-resistant cancers susceptible to immunotherapy (141). JHU083, a novel glutamine antagonist, could overcome cancer immune escape and boost anti-cancer responses (139). These findings demonstrate that amino acid deprivation can cause immunosuppressive TME, decreasing anti-tumor immune responses.

Tryptophan, as another key amino acid, is required for T cell function, but indoleamine 2,3-dioxygenase (IDO), the enzyme that catalyzes tryptophan to kynurenine, is extensively expressed in human tumors. Kynurenine, a ligand for the aryl hydrocarbon receptor (AHR), suppresses T cells’ anti-tumor response (142). IDO expression has been associated with poor prognosis by consuming tryptophan (143).

Immune suppression occurs within the TME when tumor cells, DCs, macrophages, cancer-associated fibroblast (CAF), tumor-associated macrophages (TAMs), and MDSCs express IDO (60, 144, 145). IDO prevents T cell survival, proliferation, and function by tryptophan consumption (146–148). The AHR binds to kynurenine produced by IDO, promoting immunosuppression by increasing Treg development, which inhibits anti-tumor immune response (149, 150). Furthermore, tumor cell arginine metabolism increases tumor development and immune evasion. So, targeting the IDO-kynurenine-tryptophan axis and depleting arginine in combination with anti-PD-1 could be a promising approach for eliminating immunotherapy resistance (65, 151).

### Hypoxia

Rapid tumor cell proliferation causes low oxygen concentration and hypoxic stress in the TME. At the center of the TME cellular mass, HIF-1α/β is activated in response to low oxygen levels, allowing cells to adapt to hypoxic environments (152). Proliferation, differentiation, angiogenesis, metastasis, and metabolic reprogramming of cancer cells can control by HIF (153–155). HIF is one of the pathways that can induce CD39 and CD73 expression and promotes ADO formation (**Figure 1**) (156, 157).

Hypoxia appears to be a significant metabolic regulator in TME, leading to immunosuppression or therapy resistance by inhibiting CD4^+^ T cell effector function and increasing Treg activity (158–160). Hypoxia also causes immunosuppression by increasing the expression of PD-L1 on tumor cells and immuno-modulatory metabolites such as lactate and ADO (161, 162). Nitroglycerin (also known as GTN), a nitric oxide (NO) signaling activator, and TH-302, a hypoxia-activated prodrug, in hypoxic cancer cells inhibits PD-L1 expression, decreases MDSCs and hypoxia-mediated cytotoxic T-cell death, overall, making cancer cells more susceptible to T cell-mediated cytotoxicity (162, 163).

Hypoxia can decrease anti-tumor immunity by affecting functional effector cells in TME. For example, it inhibits the killing capability of NK cells by reducing their activation receptors, CD16 and NKG2D (164). HIF-1 induced the expression of CD47, the “don’t eat me” signal, on tumor cells, leading to decreased phagocytosis and promoting cancer growth and immune evasion (165).

Moreover, HIF-1 reduces the surface expression of the major histocompatibility complex (MHC) class I chain-related (MIC) immune cell activator, allowing the tumor cell to escape immune response (166). Hypoxia-induced C-C motif chemokine ligand 28 (CCL28) promotes tumor immune evasion by attracting C-C motif chemokine receptor 10 (CCR10)-positive Tregs to the tumor site (167). Overall, hypoxia enhances the VEGF expression, which stimulates M2 macrophage polarization and MDSC infiltration and suppresses antigen presentation, DC maturation, T cell anti-tumor function, contributing to tumor progression (168–171). In addition to the hypoxic environment, tumor cells can escape immune surveillance in an acidic TME through cytokine denaturation (172, 173). As mentioned before, eATP stimulates P2X7R and P2X7R enhanced mTOR/HIF1/VEGF signaling pathway. Also, the intracellular HIF-1 was activated by stimulating the A2BR and Warburg effect. Then HIF-1α increases cancer drug resistance by increasing GLUT1 (**Figure 2**) (17, 92, 100, 101). As a result, a therapy targeting HIF-1 or reducing acidity like blocking proton export could be a fascinating strategy for increasing immunotherapy efficacy.

## Immune cell metabolism in TME and therapy resistance

FAO and OXPHOS are the primary energy sources for naive T lymphocytes and are essential for Treg cells, while glycolysis, glutaminolysis, OXPHOS, and lipid synthesis are enhanced in effector T cells (174–177). The metabolic differences between CD4^+^ T and CD8^+^ cells are significant too. Compared to CD4^+^ T cells, CD8^+^ T cells are less reliant on oxygen, OXPHOS, and GLUT1 levels in the TME and have more metabolic flexibility, causing CD8^+^ T cells to proliferate faster (178–180). Tregs in the TME are highly apoptotic because of oxidative stress, and these apoptotic Tregs promote immunosuppression by high conversion of ATP to ADO by CD39 and CD73 (181).

TAMs play an essential role in tumor cell proliferation, angiogenesis, and the development of an immunosuppressive TME. TAM’s metabolic profiles are frequently marked by increased glycolysis, FA synthesis, and glutamine metabolism. These profiles cause TAM to contribute to angiogenesis and metastasis (182–184). TAMs can release arachidonic acid, tumor necrosis factor-α (TNF-α), TGF-β, and IL-6 in melanoma, causing cancer cells to produce VEGF-A, which accelerates angiogenesis (185). Also, IL-6 through PGK1 phosphorylation in cancer cells and lncRNAs released from TAM increases tumor cell glycolysis and thereby boosts malignant progression (186, 187). TAM-derived metabolites play a significant function in chemotherapy resistance in tumors. TAM abundance is directly associated with a lower response to a chemotherapy drug, gemcitabine, in pancreatic ductal adenocarcinoma (PDAC) (188). Immunosuppressive cells like TAMs express P2X7R at high levels, and the lack of P2X7R disrupted the polarization of TAMs (189).

Exogenous lipid uptake by lipid transport receptors shifts metabolic reprogramming of cancer-infiltrating MDSC from glycolysis to FAO (190). Increased exogenous FA intake by MDSCs promotes tumor growth by increasing their immunosuppressive effect on T-cells (151, 191). By increasing NO and ROS production, depletion of L-cysteine and L-arginine, or secretion of inhibitory cytokines, MDSCs suppress T cell activation and proliferation and drive the development of macrophage M2 and Tregs (192). Furthermore, MDSCs could contribute to establishing an immunosuppressive TME, which boosts cancer development and immune escape (140).

DCs are effective stimulators of T cell activation because they absorb, process, and present antigens in the TME. Tumor-associated DCs generate ROS, which causes endoplasmic reticulum (ER) stress and lipid production. The accumulation of lipids in DCs can suppress the anti-tumor response by decreasing antigen presentation capacity (193). The ability of tumor-infiltrating DCs to present tumor antigens modifies due to lipid accumulation and reduced arginine and tryptophan, affecting T cell anti-tumor immunity and resulting in tumor immune escape (194, 195).

Tumors can evade immune surveillance due to ADO-AR signals, which decrease the anti-cancer function of immune cells like CD8^+^ T cells, DCs, NK cells, and M1 macrophages while boosting the immunosuppressive function of cells like MDSCs and Tregs, increasing the expression of IL-10, TGF-β, arginase-2, and IDO-1, lead to Th2 or M2 differentiation (196). In a mouse model, treatment with the CD39 inhibitor increased CD8^+^ T cells and NK cell-mediated killing capacity, indicating improved anti-tumor immunity (197). Furthermore, some nucleotides, e.g., ADO, and cancer-derived amino acids, e.g., kynurenine, affect immunotherapy efficacy. To overcome treatment resistance, multiple blockers targeting these compounds can be combined with PD-1 or PD-L1 blockers (151). In tumor models, the combination of anti-CD73 and anti-PD-1 antibodies exhibited synergistic effectiveness against ADO-driven immunosuppression, prompting a phase I trial of anti-CD73 in cancer patients (NCT02503774) (198).

Because ADO in the TME impairs the immune response, the CD39/CD73 and ADO receptors are considered promising anti-cancer therapeutic targets. Chemotherapy and radiotherapy cause tissue damage and cell death, resulting in ATP release, CD73 overexpression, and increased ADO in the TME, so CD73 inhibition combined with radiotherapy and ICPI improved DC infiltration and T cell responses (199, 200).

## Strategies for combating drug resistance

Single-drug treatment approaches kill sensitive tumor cells while allowing resistant cancer cells to survive and proliferate, so these approaches are most likely to fail due to drug resistance. But, combination therapy, which targets more driver genes simultaneously or with energy blocking, suppresses more clones in a tumor and improves the efficacy of the drug. Novel therapeutic approaches, “on and off” and “high dose followed by low dose”, lead to extended survival and inhibit drug resistance since this intermittent or flexible dosing permits the competition of sensitive and resistant cells and prevents the formation of drug-resistant cells (201). These findings prompted clinical trials of this intermittent dosage strategy (NCT02196181).

Cancer cells could circumvent any strategy, but they couldn’t escape the energy demand for their growth, proliferation, metastasis, or drug resistance. Tumor cells tend to have greater ATP levels for survival and therapy resistance. Limiting the energy supply of cancer cells is one approach to overcoming resistance. Prescreening tumors based on their ability to internalize ATP and the expression of a certain set of ABC transporters will give meaningful insights for selecting appropriate anti-cancer drugs and predicting patient response to therapies, restricting drug resistance, and improving therapeutic effectiveness. Drug efficacy may be improved by lowering eATP concentrations, ATP synthesis-inhibitor, or preventing ATP internalization *via* diminishing purinergic receptor activation, causing tumor cells to stop growing and cell death (51). When injected into a rat glioma model, an ATPase called apyrase was found to inhibit the growth of glioblastoma (70).

The signaling of purinergic receptors appears to contribute to cancer progression and resistance to treatment by altering the metabolism of cancer cells. Therefore, therapeutic interventions that are considered to inhibit cancer cell metabolism and purinergic receptors simultaneously can potentially reduce resistance to treatment. Given that, molecules such as the P2X7R have emerged as an attractive target for anti-tumor therapy in various tumors. Also, P2X7R is an aerobic glycolysis potent activator (18). The loss of the P2X7R through glucose intolerance, insulin resistance, and decreased FA metabolism reduce therapeutic resistance (127).

Humans express functional P2X7R splice isoforms lacking the C terminal domain, like P2X7R variant B, a variant incapable of forming the macropore and lacking cytotoxic activity but yet possessing ion channel features (25, 202). Interestingly, full-length P2X7R variant A facilitates doxorubicin and daunorubicin cellular absorption and cytotoxicity. In contrast, the P2X7RB has tumor-promoting properties and is correlated with a poor prognosis in a variety of cancers (17, 203–205). The P2X7RB protects cells from daunorubicin toxicity, most likely because of a daunorubicin-dependent rise in ATP concentration in the TME. As a result, treatment with daunorubicin increased P2X7RB expression while decreasing P2X7RA expression in AML patients, leading to P2X7RB overexpression. So, the P2X7RB is a promising therapeutic target for this leukemia (26).

Although the normal cells are more flexible in consuming different energy sources, tumor cells tend to be inflexible. For instance, aggressive tumors with a poor prognosis are glucose-dependent, according to positron emission tomography (PET) imaging (206). This difference between tumor and non-tumor cells can be investigated to tackle cancer growth. These tumor cells are addicted to glucose and are more sensitive to environment glucose concentration than non-tumor cells, so dying faster in glucose-depleted conditions (207). Glycolysis enzymes (GAPDH and LDH) inhibitors such as 3-bromopyruvate, FX11, and oxamate, which reduce intracellular ATP levels, may make tumor cells more susceptible to anti-cancer therapies (208, 209). Using a glucose transport inhibitor, a glycolysis inhibitor, eATP degrader, or inhibition of eATP internalization in combination with other therapies may be especially effective in triggering tumor cell death.

## Perspectives

Stemness, tumor metastasis, and therapeutic resistance all require metabolic reprogramming. However, the TME has a complicated composition, and the metabolic landscape inside this microenvironment is poorly known; the TME metabolite’s signature or pattern could be a potential cancer biomarker. On the other hand, developing metabolic inhibitors to maintain T cell activity is critical. As a result, focusing on the cancer cell, metabolic pathways will not only increase our understanding of the cellular interplay in the TME but will also let us overcome immunotherapy resistance by broadening our therapeutic options. Potential metabolic blockers that can be employed in combination with ICPIs will aid in the discovery of new anti-cancer immunotherapy.

Fighting therapeutic resistance appears to be an endless battle because tumor cells can always explore novel strategies to combat present treatment. Identifying novel targets for developing anti-cancer therapies are becoming increasingly important to combat chemotherapeutic resistance. Also, the induction of a specific type of cell death called ICD can help to suppress resistance to treatment in tumor cells (perhaps by altering tumor cell metabolism). Last but not least, the well-explained function of ATP in ICD gives room for a different treatment approach that aims to trigger a controlled release of ATP in the TME to increase DC responses. A combination therapy involving ICD inducers and immunotherapy (e.g., anti-PD-L1), which induces a long-lasting immune response, paints a promising prospect in reducing therapy resistance.

Recent research attempts to determine how the P2X7R affects cancer cell metabolic reprogramming, e.g., intracellular ATP synthesis, allowing cell division and cytoskeletal alterations required for tumor development and metastasis. To improve current anti-tumor therapeutic strategies, it is crucial to comprehend the significance of the complex metabolic changes related to cancer and a deep understanding of P2X7R expression, regulation, and involvement in metabolic disorders, cancer metabolism, and metabolic reprogramming.

## Conclusion

In this review, we investigated the role of purinergic receptors in the development of resistance to therapy through changes in tumor cell metabolism. For cancer progression and therapeutic resistance, metabolic reprogramming is necessary. Increased energy demands of tumor cells, or a lack of nutrients or oxygen, might cause metabolic changes that affect cell fate. Cancer cells are exposed to a combination of receptors and extracellular molecules, and their combination determines the cell fate. To develop and win, cancer hijacks the whole body’s energy and function. Cancer cells increase nutrient uptake, deplete oxygen, elevate TME acidity, and increase the pro-tumor metabolic pathway to generate an immunosuppressive TME that supports cancer proliferation and immune escape.

The purinergic receptors are crucial in both normal and malignant cells. They contribute to therapy resistance by modifying cancer cells’ glucose, lipid, and amino acid metabolism. Extensive *in vitro* and *in vivo* preclinical findings showed that extracellular nucleotides and their receptors affect tumor progression. In recent years, mechanisms for ATP release into the extracellular milieu and plasma membrane receptors responsible for these various effects have been defined. An important issue that exists is the dual roles of ATP and signaling caused by purinergic receptors, which can lead to an increase or decrease in resistance to treatment. Although factors such as ATP concentration, type of binding receptor, or tumor stage can affect the final result, there is a strong need for more extensive studies on why these dual behaviors of ATP and purinergic receptors exist. Therefore, to improve current anti-tumor medicines, a thorough understanding of purinergic receptor expression, regulation, and role in cancer metabolic reprogramming is crucial.

## Author contributions

All authors contributed to the study conception and design. Review and editing were performed by MR, AD,GN, BM, MB, and AT. Visualization was performed by SN, and the project was supervised by MV and YM. The first draft of the manuscript was written by HA, and all authors commented on previous versions of the manuscript. All authors read and approved the final manuscript.

## Conflict of interest

The authors declare that the research was conducted in the absence of any commercial or financial relationships that could be construed as a potential conflict of interest.

## Publisher’s note

All claims expressed in this article are solely those of the authors and do not necessarily represent those of their affiliated organizations, or those of the publisher, the editors and the reviewers. Any product that may be evaluated in this article, or claim that may be made by its manufacturer, is not guaranteed or endorsed by the publisher.
